# Supplementary material for: The Management of Acute Colonic Diverticulitis in the COVID-19 Era: A Scoping Review
Source: Medicina (Kaunas). 2021 Oct 18;57(10):1127. doi: 10.3390/medicina57101127 (PMC8538273; doi:10.3390/medicina57101127)
Supplement: Supplementary file 1 [file medicina-57-01127-s001.zip › medicina-1395305-supplementary.pdf]

Table S1. Excluded full texts (not reported data about acute diverticulitis).

Author	Journal	Published Online
Collard	Colorectal Disease	16 June 2020
Fu	Br J Surg	3 June 2020
Changzheng	ANZ J Surg	28 May 2020
Bonapasta	J Trauma Acute Care Surg	21 May 2020
Wakam	Int J Surg	19 May 2020
Coppola	Int J Surg	19 May 2020
Shanker	Int J Surg	19 May 2020
Cano-Valderrama	Br J Surg	14 May 2020
Pata	J Trauma Acute Care Surg	13 May 2020
Yao	Dis Colon Rectum	13 May 2020
Yeo	Br J Surg	7 May 2020
Huda	Int J Surg	4 May 2020
Liu	Int J Surg	4 May 2020
Tan	Ann Coloproctol	30 April 2020
Hogan	Br J Surg	24 April 2020
Faccincani	Eur J Trauma Emerg Surg	20 April 2020
Coimbra	Eur J Trauma Emerg Surg	20 April 2020
Gao	Ann Surg	13 April 2020
Liu	Indian J Surg	11 April 2020
Ahmed	J Am Coll Surgeon	9 April 2020
Slim	J Visc Surg	7 April 2020
Gallo	J Invest Surg	5 April 2020
Lisi	Dis Colon Rectum	30 March 2020
Kurihara	J Trauma Acute Care Surg	18 March 2020
Pellino	Dis Colon Rectum	17 March 2020
